# Eating Disorders

**DOI:** 10.1192/j.eurpsy.2022.63

**Published:** 2022-09-01

**Authors:** J. Treasure

**Affiliations:** King’s College London, Department Of Psychological Medicine, London, United Kingdom

## Abstract

Covid had a profound impact on services for eating disorders because of the huge increase in demand. Nevertheless, services quickly adopted a virtual form of working. This rapid change in prevalence was attributed to a number of factors, including a general increase in fear and fragmented social functioning, with a specific accentuation of higher risk associated with body weight. Recent advances in the conceptualisation of eating disorders include a move from a transdiagnostic to a more to a personalised approach. For example, it has been further demonstrated that reducing the duration of untreated illness within three years of onset is associated with a better outcome (1). This has led to the rollout of FREED, an early intervention service in the UK. Genetic associations have been compared and contrasted across the spectrum of eating disorders. People with binge eating disorders share a profile with those at risk of metabolic syndrome whereas people with anorexia nervosa have the opposite profile (2). This reconceptualization of eating disorders as conditions with both brain and body underpinnings has led to new treatment approaches. For example, there have been small proof of concept studies in which metreleptin has been administered. These show promise with rapid reductions in depression and other symptoms (3, 4). Meanwhile the value of “experts by experience,” in co designing and delivering services is an area of active investigation which offers the much needed potential of improving treatment outcomes (5).

**Disclosure:**

No significant relationships.

